# Microtomographic morphometry of the stapedius muscle and its tendon

**DOI:** 10.1007/s12565-019-00490-6

**Published:** 2019-05-20

**Authors:** Tomasz Wojciechowski, Tymon Skadorwa, Jean-Gualbert Nève de Mévergnies, Kazimierz Niemczyk

**Affiliations:** 1 Department of Otolaryngology, The Medical University of Warsaw, 1a Banacha St., 02097 Warsaw, Poland; 2 Department of Descriptive and Clinical Anatomy, The Medical University of Warsaw, 5 Chalubinskiego St., 02004 Warsaw, Poland; 3 Department of Pediatric Neurosurgery, Bogdanowicz Memorial Hospital for Children, 4/24 Nieklanska St., 03924 Warsaw, Poland; 4 Université Catholique de Louvain, 1. Place de l’Université, 1348 Louvain-la-Neuve, Belgium

**Keywords:** Stapedius muscle, Stapedius tendon, Facial nerve, Retrotympanum, MicroCT

## Abstract

The aim of this study was to evaluate the morphology of the stapedius muscle and its tendon with the use of microCT and to describe their anatomic relationship with facial nerve and incudostapedial joint. The study was performed on 16 fresh cadaveric temporal bones scanned in microtomography (microCT). Stapedius muscle and its tendon were identified in each set of images. The length of the medial and lateral border of the stapedius tendon (STL-med, STL-lat), width at the insertion to stapes (STW-s), at the point it emerges from the pyramidal eminence (STW-p) and in the half way from the pyramidal eminence to stapes (STW-m), and the length and the width of the belly of stapedius muscle (BSML and BSMW) were measured in modified axial plane. The shortest distance between the facial canal and incudostapedial joint (FN-isj), and between the facial canal and stapedius tendon (FN-st) were measured in the Pöschl plane. The average values of all distances measured were: STL-lat 1.29 ± 0.50 mm, STL-med 1.27 ± 0.44 mm, BSML 2.98 ± 0.51 mm, STW-s 0.47 ± 0.10 mm, STW-p 0.46 ± 0.12 mm, STW-m 0.35 ± 0.12 mm, BSMW 1.26 ± 0.29 mm, FN-isj 1.72 ± 0.33 mm, FN-st 1.35 ± 0.30 mm. The stapedius muscle complex consists of the tendon and the belly, and the border between them in microCT scans is not always evident. The distance between the facial nerve and the incudostapedial joint is greater than the distance between the facial nerve and the stapedius muscle tendon.

## Introduction

The stapedius muscle is usually referred to as the smallest muscle in the human body. A first detailed description of this structure and its tendon were delivered by Paaw (1615) in the early seventeenth century. At that time, researchers were aware of the existence of three ossicles in the ossicular chain but they investigated the possibility of the presence of sesamoids in the area of the middle ear, particularly inside the tendons of the stapedius and tensor tympani muscles. Late in the fourth decade of the seventeenth century, Vesling (1651) and his successor Folius (1645) published an anatomic description of the stapedius tendon in which they tried to prove the presence of a fourth auditory ossicle. Despite the fact that an additional bone exists within the stapedius tendon in other species, its presence in humans has not been confirmed in either cadaveric or radiologic studies (Asherson 1978; Graboyes et al. 2011).

The development of stapedius muscle and its tendon has been the subject of very few articles, and even then, they refer to animals rather than to humans (Arensburg et al. 1981; Borg et al. 2009; van den Berge and van der Wal 1990). In the last 60 years, since Shea (1958) first introduced the procedure of stapedotomy, surgery of the stapes has evolved significantly. Nowadays, several authors still wonder whether to preserve the stapedius tendon during this procedure (Colletti et al. 1988; Gros et al. 2003) but not a single paper refers to the tendon morphology. It is striking that the professional literature still lacks a detailed morphometry of stapedius muscle and the description of its relationship to surrounding structures in humans, especially to the facial nerve, which can overhang the area of oval window and cause problems when accessing the footplate of the stapes during intrameatal approach for stapedotomy.

The introduction of microtomography (microCT) in 1990s of the twentieth century enabled detailed studies of the structures that could not be fully visualized in conventional high resolution computed tomography (HRCT) (Li et al. 2018). The aim of this study was to evaluate the stapedius muscle and its tendon with the use of microCT. The findings can be of value for the understanding of stapedius muscle morphology and its relationship to adjacent structures.

## Material and methods

The study was performed on 16 fresh cadaveric temporal bones provided and anonymized by the Department of Forensic Medicine, Medical University of Warsaw. Initially, all specimens were labeled with a side and a number and prepared in order to fit into the microCT scanner. External auditory meatus, middle ear spaces and cochlea were intact in every temporal bone. All the scans were obtained with the scanner Phoenix Xray (GE Sensing & Inspection Technologies, Wunstorf, Germany) with parameters: voxel dimensions 0.07 × 0.07 × 0.07 mm; the exposition performed with source voltage of 120 kV and current of 70 mA. All scans were analyzed in RadiAnt DICOM Viewer 4.0.3 (64-bit) and data obtained were analyzed statistically with the use of StatSoft Statistica 13.1 software.

**Fig. 1:**
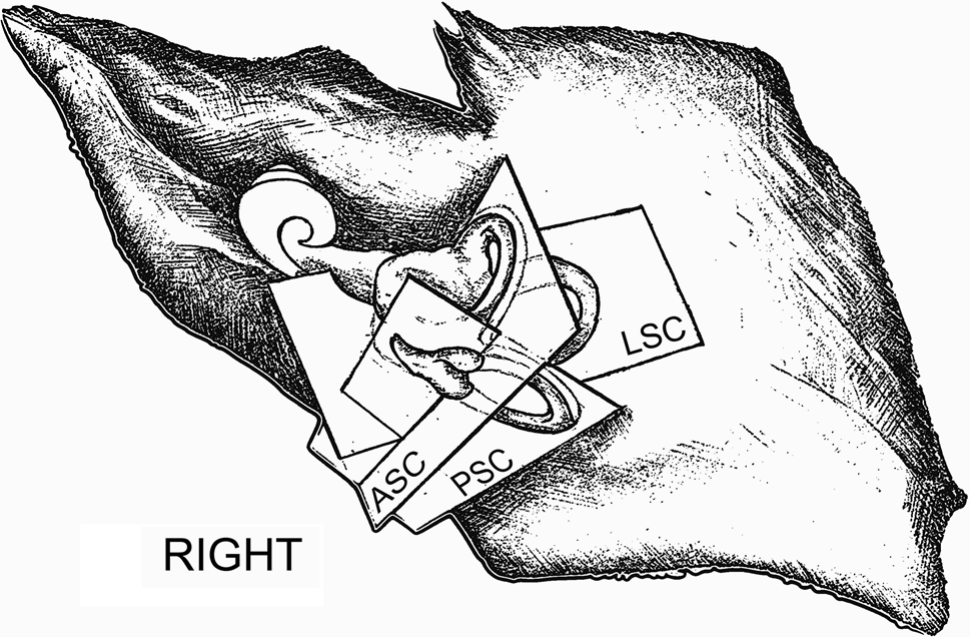
Reference planes used in the study: *ASC* anterior semicircular canal, *LSC* lateral semicircular canal, *PSC* posterior semicircular canal

**Fig. 2:**
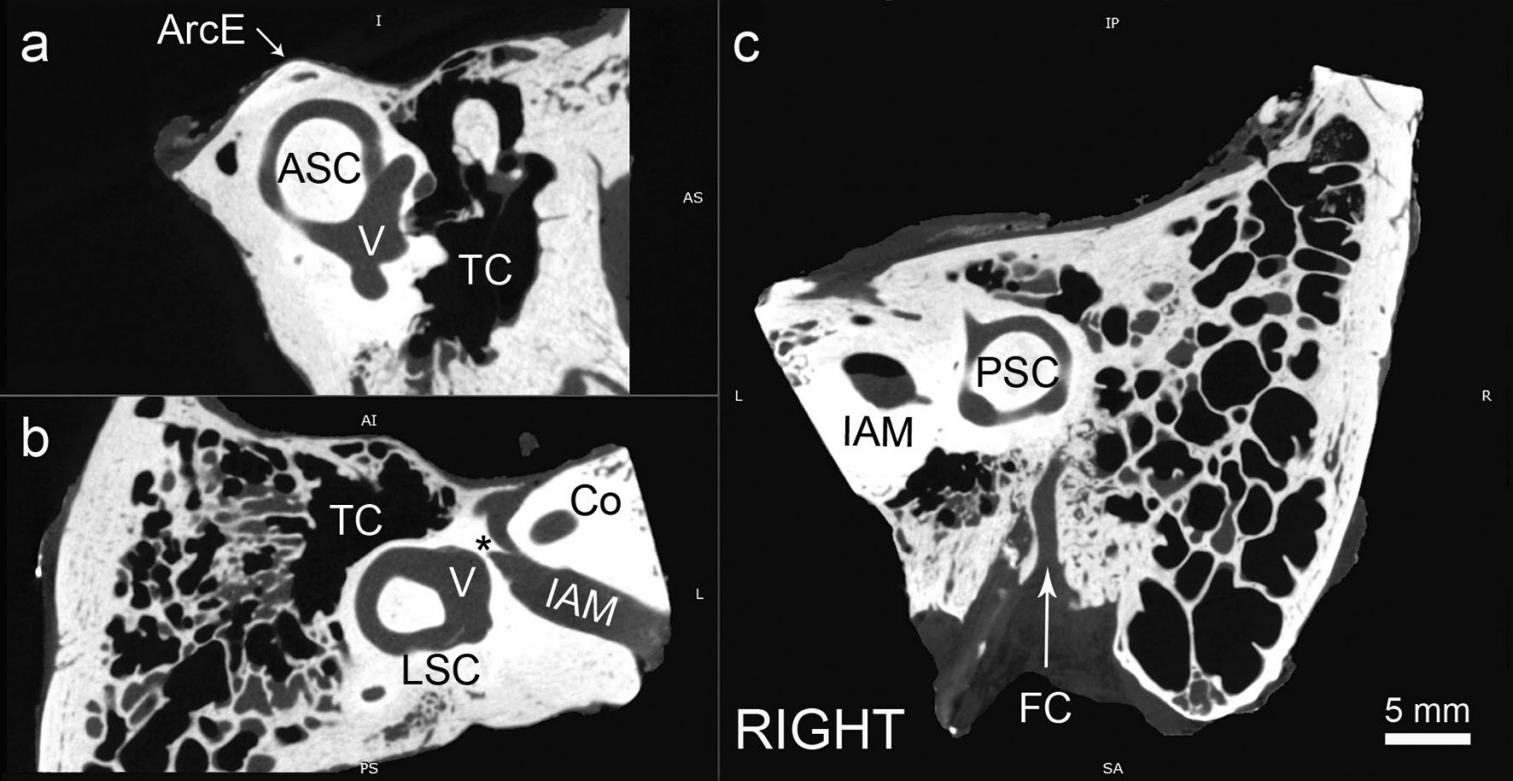
Planes used in the study for identification of the stapedius muscle. **a** Pöschl plane (anterior semicircular canal); **b** lateral semicircular canal plane; **c** posterior semicircular canal plane; *ASC* anterior semicircular canal, *ArcE* arcuate eminence, *TC* tympanic cavity, *LSC* lateral semicircular canal, *Co* cochlea, *IAM* internal acoustic meatus, *PSC* posterior semicircular canal, *FC* facial canal (canal for facial nerve), *V* vestibule, *asterisk* Bill’s bar

In the first step of the analysis, the three reference planes were established. We used the planes of three semicircular canals: anterior (ASC), lateral (LSC) and posterior (PSC) (Fig. 1). The first plane was described by Pöschl (1943) and in our study was the most important. In this plane, laterally to the anterior semicircular canal (ASC), the tympanic cavity (TC) can be seen. The ossicular chain has its characteristic ‘molar tooth appearance’ and incudostapedial as well as incudomalleal joints are clearly visible. The oval window with stapes is just inferior to the facial canal (Fig. 2a). The second plane was a modified axial view tilted to be parallel to lateral semicircular canal. In such a view, LSC presents a classic ‘signet ring appearance’ (Fig. 2b). In addition, the Bill’s bar (asterisk) at the fundus of IAM can also be spotted. The third plane was a consequence of the remaining settings—the PSC and the common crus can be seen with clarity, laterally to the IAM (Fig. 2c).

**Fig. 3:**
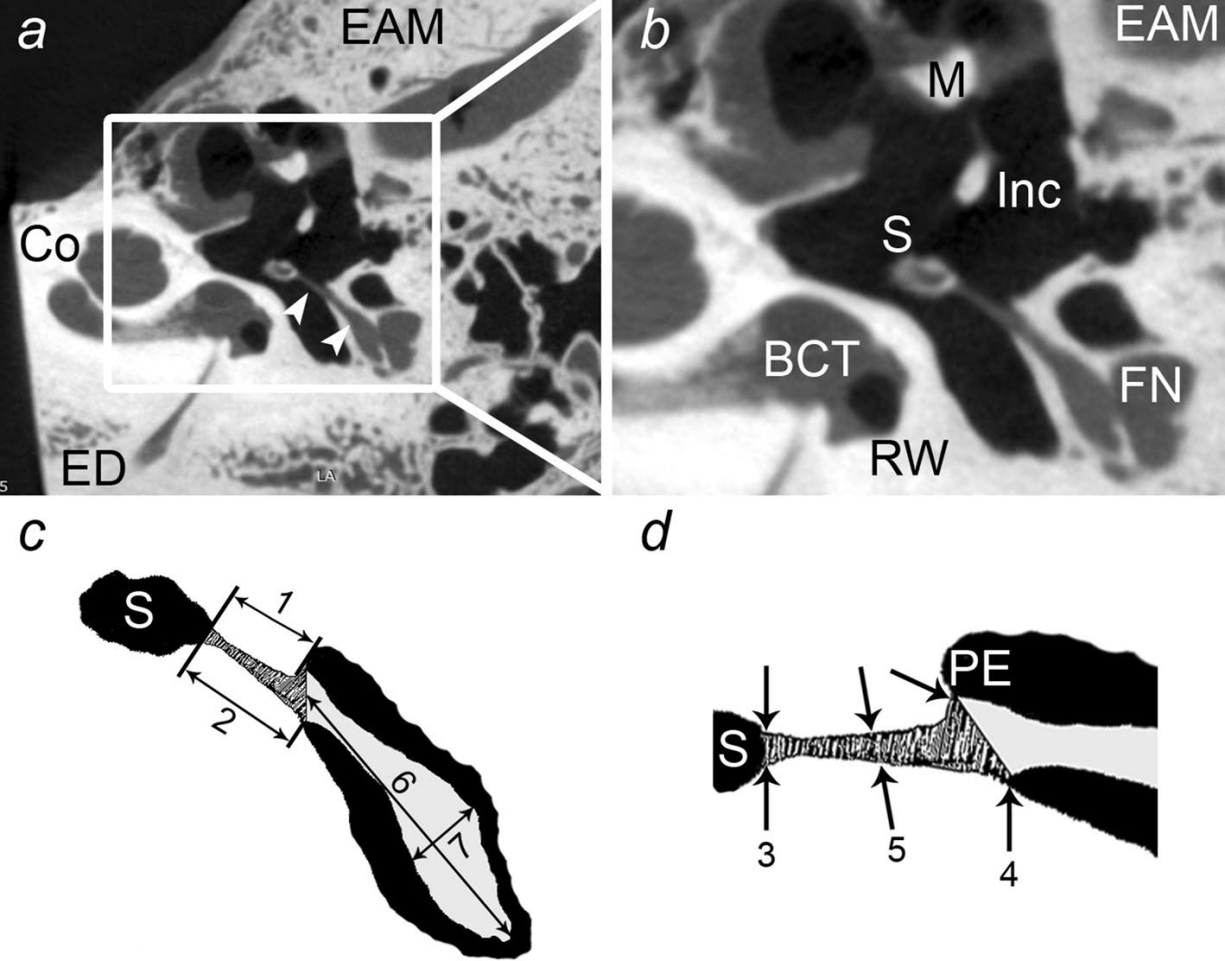
**a** The location of the right stapedius muscle (*white arrowheads*) and adjacent structures: *Co* cochlea, *ED* endolymphatic duct, *EAM* external acoustic meatus. **b** Detailed view shows structures critical in stapedotomy: *S* head of stapes, *Inc* incus, *M* malleus, *FN* facial nerve, *BCT* basal cochlear turn, *RW* round window, *EAM* external acoustic meatus. **c** Schematic view of the stapedius muscle (*dashed pattern* tendon,* grey* belly) with parameters described in the text:* S* head of stapes;* 1* SML-lat;* 2* SML-med;* 6* BSML;* 7* BSMW. **d** The measurements of the tendon of stapedius muscle:* S* head of stapes;* PE* pyramidal eminence;* 3* STW-s;* 4* STW-p;* 5* STW-m

**Fig. 4:**
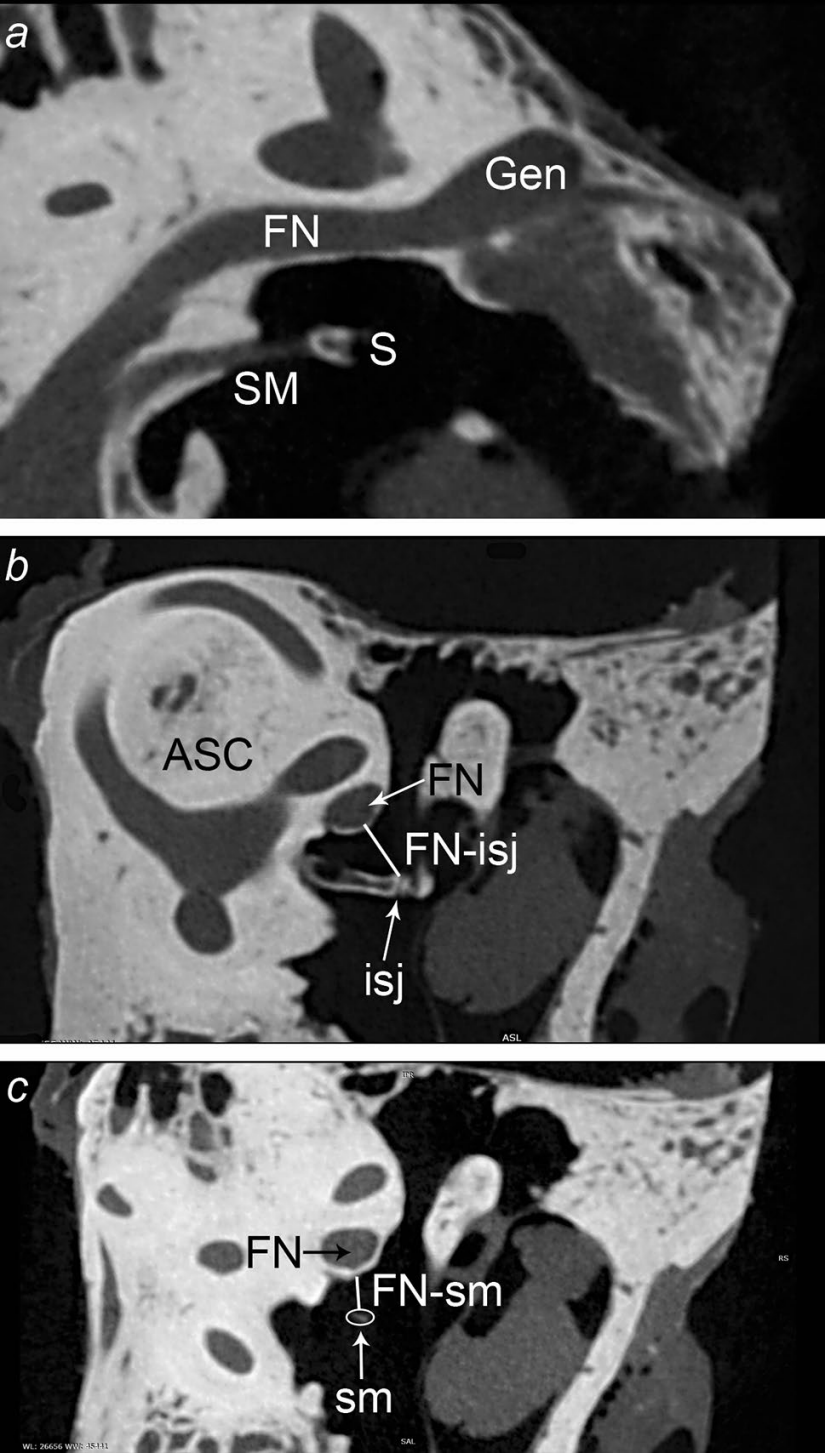
Anatomical relationship between the stapedius muscle and facial nerve. **a** General view: *SM* stapedius muscle, *S* head of stapes, *FN* facial nerve, *Gen* geniculate ganglion. **b**, **c** Parameters measured in the plane of ASC: *ASC* anterior semicircular canal, *FN* facial nerve, *isj* incudostapedial joint, *sm* tendon of stapedius muscle (transverse cut); FN-isj, *FN-st* parameters described in the text

The next step was to identify ST in the plane of the lateral semicircular canal, and to measure its length along its medial and lateral border (STL-med, STL-lat), width at the insertion to stapes (STW-s), at the point it emerges from the pyramidal eminence (STW-p) and at the half way point from the pyramidal eminence to stapes (STW-m). In the same plane, the length and the width of the belly of stapedius muscle were measured (BSML and BSMW, Fig. 3). Then we were able to identify the tympanic part of the facial nerve on the scans parallel to anterior semicircular canal. After the identification, the shortest distance between the facial canal and incudostapedial joint (FN-isj) and between the facial canal and stapedius tendon (FN-st) were measured (Fig. 4). All the measured parameters with abbreviations are presented in Table 1. Appropriate data were placed in the study database and underwent statistical analysis.

**Table 1:** Abbreviations used in the study

Abbreviation	Explanation
STL-lat	Stapedius tendon length-lateral border
STL-med	Stapedius tendon length-medial border
STW-s	Stapedius tendon width-stapes
STW-p	Stapedius tendon width-pyramidal eminence
STW-m	Stapedius tendon width-middle
BSMW	Belly of stapedius muscle width
BSML	Belly of stapedius muscle length
FN-isj	Facial nerve-incudostapedial joint
FN-st	Facial nerve-stapedius tendon

## Results

The average length of the stapedius tendon at its lateral border was 1.29 ± 0.50 mm (0.73–2.42 mm) and 1.27 ± 0.44 mm (0.60–2.18 mm) at its medial border. The difference between these two parameters was not statistically significant (*t* test; *P* = 0.89).

**Fig. 5:**
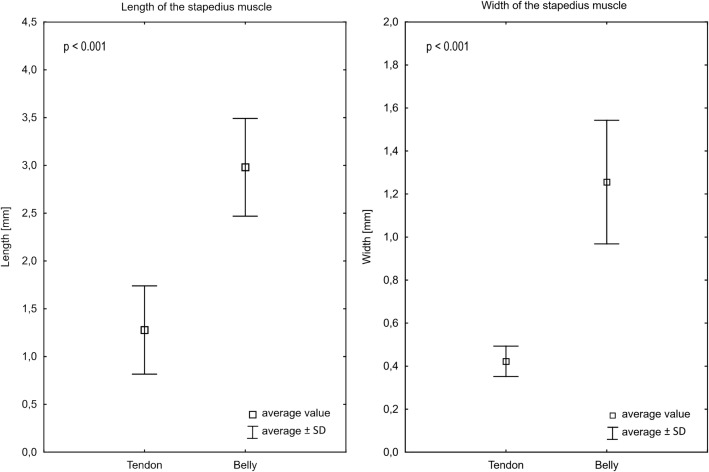
Descriptive statistics of the stapedius muscle

The average length of the belly of the stapedius muscle was 2.98 ± 0.51 mm (2.19–4.01 mm). The difference between the length of the tendon and the belly is presented in Fig. 5.

The width of the stapedius tendon was the greatest at the level of two attachments: at the stapes it was 0.47 ± 0.10 mm (0.28–0.60 mm) and at the pyramidal eminence it was 0.46 ± 0.12 mm (0.29–0.73 mm). These values were not significantly different (*t* test; *p* = 0.85). The width at half way between two attachments was smallest (K–W test; *p* = 0.0014) at 0.35 ± 0.12 mm (0.25–0.73 mm). The average width of the belly was 1.26 ± 0.29 mm (0.78–1.67 mm). The relationship of the width of the belly and a tendon is shown in Fig. 5.

The average distance between FN and the incudostapedial joint was 1.72 ± 0.33 mm (the shortest 1.20 mm and the greatest 2.23 mm) and between the FN and the ST was 1.35 ± 0.30 mm (0.91–1.75 mm). The difference between them was statistically significant (*t* test; *p* = 0.002).

Detailed values of measured parameters are presented in Table 2.

**Table 2:** Direct parameters measured in the study (*LSC* measurements in the plane of lateral semicircular canal,* ASC* measurements in the plane of anterior semicircular canal, abbreviations explained in Table 1)

No.	LSC (mm)								ASC (mm)	
	STL-lat	STL-med	STW-s	STW-p	STW-m	BSMW	BSML	STL + BSML	FN-isj	FN-st
1	1.07	0.89	0.54	0.41	0.32	1.67	3.70	4.77	1.20	1.42
2	0.86	0.99	0.51	0.43	0.25	1.54	2.82	3.81	1.69	1.04
3	2.04	1.92	0.41	0.73	0.29	1.53	4.01	6.05	2.16	1.65
4	0.79	1.06	0.59	0.63	0.50	1.36	2.19	3.25	1.45	1.14
5	2.42	2.18	0.32	0.34	0.73	1.10	2.96	5.38	2.20	1.58
6	1.34	1.31	0.49	0.32	0.28	1.42	3.73	5.07	1.50	1.13
7	1.81	1.36	0.53	0.55	0.26	1.03	2.56	4.37	1.65	1.75
8	1.50	1.25	0.42	0.44	0.27	0.78	3.08	4.58	1.61	1.75
9	0.77	0.60	0.60	0.51	0.42	1.20	3.05	3.82	1.55	1.60
10	1.15	1.33	0.44	0.29	0.27	1.49	2.57	3.9	2.23	1.45
11	0.78	0.80	0.55	0.49	0.33	0.81	3.17	3.97	1.50	1.00
12	1.33	1.37	0.49	0.39	0.33	1.51	2.51	3.88	2.14	1.05
13	0.99	1.09	0.28	0.38	0.31	0.93	2.73	3.82	1.39	0.91
14	1.29	1.69	0.40	0.40	0.32	1.26	3.31	5	1.85	1.55
15	1.76	1.71	0.55	0.57	0.37	1.50	2.89	4.65	1.95	1.53
16	0.73	0.72	0.32	0.44	0.27	0.96	2.40	3.13	1.40	1.00
Average	1.29	1.27	0.47	0.46	0.35	1.26	2.98	4.34	1.72	1.35

## Discussion

Given its complexity, the temporal bone has always been a challenge for medical researchers, especially in terms of diagnostic imaging. With the development of modern techniques it became obvious that obtaining a proper image of the crucial structures of internal ear and tympanic cavity, such as ossicular chain, facial nerve canal or middle ear muscles, is not possible with the use of standard anatomic planes as a reference (Ozgen et al. 2008; Chen and Mafee 2014). This conclusion contributed, in the last century, to the establishment of new specific reference planes for the visualization of particular structures in the temporal bone. Defined by Pöschl (1943), a plane of the anterior semicircular canal is often used to identify the tympanic part of the facial canal (as most of potential dehiscence are observed in this segment), but also for oval and round windows, incudostapedial joint and stapedius muscle tendon. An axial plane is of use when one wants to visualize the incudomalleal joint, but in standard temporal bone imaging, this plane is tilted so it runs parallel to the axis of the lateral semicircular canal. In this plane, a researcher can find the tympanic sinus, pyramidal eminence, or facial nerve with geniculate ganglion. Taking this into consideration, we would always encourage making a multiplanar reconstruction for the purpose of deepened research of the temporal bone, especially with suspicion of disease.

The stapedius muscle with its tendon, although known for years, had not been yet described in detail, especially in humans. Radiological attempts to describe this muscle had encountered technical obstacles, such as the resolution of CT scanners, resulting in the fact that the stapedius muscle was often referred to as the space represented by the bony canal or excavation. Besides, many of the references in classical anatomic atlases turned out to be a dead end in terms of measurements. Unur et al. (2002) provide data of ossicular chain measurements taken with the aid of micrometer—malleus and incus were of average length 7.69 mm and 6.47 mm, respectively, and stapes height was 3.22 mm—all of these are greater than the length of stapedius muscle tendon in our study; in particular, the height of the stapes is more than two times greater. Furthermore, Unur et al. (2002) conclude that the values are similar to those of previous studies. Fang et al. (2013) identified five types of stapedius muscle—the typical single canal was labeled as ‘general type’ and any different course was classified as a variation named ‘dilated type’, ‘enwrapped type’, ‘bifurcated type’ or ‘mixed type’. We have not encountered any variations of the structures mentioned above in our material. The osseus canal was similar in all specimens, and was a sole chamber always localized medially to the mastoid portion of the facial nerve, and did not divide on its course. In all our cases, the tendon inserted to the neck of stapes. In this light, one must be advised that the canal of the belly of the stapedius muscle may be damaged when accessing the tympanic sinus during canal wall up tympanoplasty with a retrofacial approach.

The belly of the stapedius muscle is a dominant portion of the muscle. The measurements revealed a remarkable difference in the length and width of the tendon and the belly. The average length of the tendon was significantly lower than the average length of the belly (almost twice). The belly was also significantly wider, with an average width 2.5 × higher than the tendon. For the purposes of measurements, we established a sharp border between these two elements of the muscle at the level of pyramidal eminence. However, in practice, the border between them is not so well demarcated, as during the stapes surgery only the tendon can be seen. This fact brings us to conclusion that the entire stapedius muscle complex, including the tendon, has an average length of 4.25 ± 0.75 mm (3.12–5.96 mm). This value may be a reference for future descriptions (i.e., radiologic). The new term stapedius muscle complex, as proposed in our study, refers to the whole stapedius (muscle) but should be used only when describing CT scans. In the images, both belly or tendon have the same density, i.e., there is no difference in color to find the real point where the tendon originates. What is more, either ossified or shortened stapedius tendon were discussed by Zawawi et al. (2014) as a possible cause of conductive hearing loss. The present findings may be of use when establishing normative values of the length of the stapedius tendon.

**Fig. 6:**
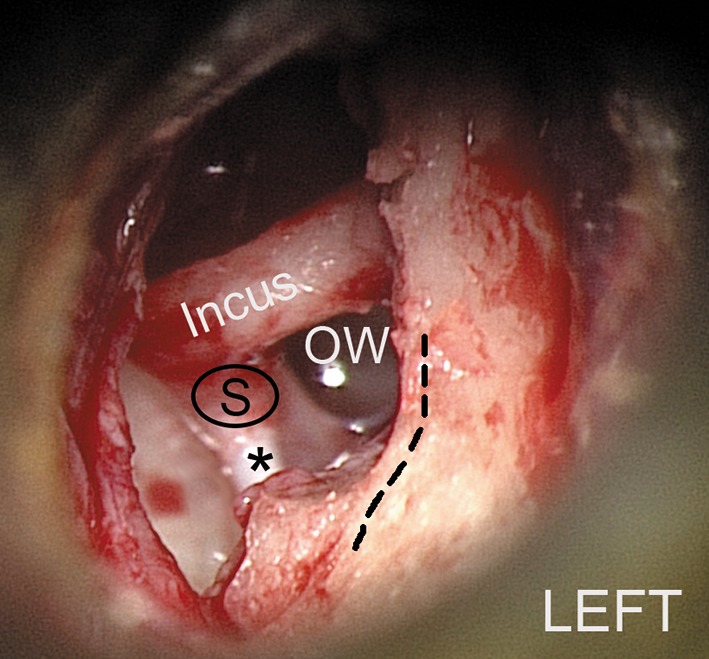
Intraoperative view of the left stapedotomy. Note the location of the stapedius tendon (*asterisk*) attaching to the head of stapes (S). *OW* Oval window,* dashed line* course of the facial nerve

Comparison between the measured parameters may be of value also for surgical purposes. The distance between FN and the incudostapedial joint was comparable with the average length of the tendon—the only visible portion of the stapedius muscle during the stapes surgery. In our material, the greater the length of the tendon, the more this parameter approached the total distance between FN and incudostapedial joint. This distance was more constant than the length of the tendon, which was characterized by a larger range of measured values. According to the literature, the variety of the course of the facial nerve in its tympanic segment is the most important matter that has to be taken into consideration when planning stapes surgery (Tccar et al. 2000; Silverstein et al. 1998). A surgeon should be aware not only of the anatomic conditions inside the middle ear, but also of possible variations and the distances between crucial structures on the way to the oval window. In order to estimate how much space there is to operate, we measured the shortest distance from the facial nerve to the incudostapedial joint as well as from the facial nerve to stapedius muscle tendon. The first of these two distances was longer (1.72 ± 0.33 mm) and it was statistically significant. This observation may provide information on what kind of surgical tools are expected to be used during surgery as well as making the decision what sort of prosthesis may be used under different anatomic conditions. However, we cannot compare our data because of the lack of information on this topic in the literature. In our opinion, the fact that the FN-isj distance is greater than FN-st may indicate that the corridor to the oval window and stapes widens from posterior to anterior. The region of posterior crus of stapes may not only be concealed at first by the posterior wall of the external acoustic meatus but also by the stapedius muscle tendon in close relationship to the angle between the tympanic and mastoid part of facial canal (Figs. 4, 6).

During surgical approach to the area of the stapes footplate, a surgeon encounters a few obstacles. When the tympanomeatal flap is made, the chorda tympani is retracted and, in some cases, the posterosuperior aspect of the external auditory meatus is removed, and the surgeon can see the incudostapedial joint, stapes with stapedius muscle and oval window (Fig. 6). The anatomical conditions may vary—the facial nerve may partially cover the stapes, and a spatial configuration between the structures mentioned above can be unfavorable for the surgeon (Shea 1958; Colletti et al. 1988). Similar problems may occur during cochlear implantation. Roberson et al. (2006) pointed out that, in some cases of low emergence of stapedius tendon, it has to be cut in order to perform cochleostomy safely and accurately. One may be advised only if the possible variations are known and CT scans are analyzed before the surgery. MicroCT analysis might possibly provide an instrument for surgical imagination.

Our study has a few limitations. First, we were unable to gather information about the gender and age of the specimens. This information would allow analysis of correlation between the dimensions of stapedius muscle in male and female populations and across ages. Further studies are also needed to describe developmental morphology of the stapedius muscle in fetuses and children, and compare it to the adult population.

## Conclusions

1. Visualization of fine structures inside the petrous part of the temporal bone requires more reformats than in standard planes only.
2. The stapedius muscle complex consists of the tendon and the belly, and the border between them in microCT scans is not always evident.
3. The distance between the facial nerve and the incudostapedial joint is greater than between the facial nerve and stapedius muscle tendon.
4. MicroCT provides adequate images for anatomic and functional studies of stapedius muscle complex.
